# Therapeutic Effects of Botulinum Toxin A, via Urethral Sphincter Injection on Voiding Dysfunction Due to Different Bladder and Urethral Sphincter Dysfunctions

**DOI:** 10.3390/toxins11090487

**Published:** 2019-08-23

**Authors:** Yu-Khun Lee, Hann-Chorng Kuo

**Affiliations:** Department of Urology, Hualien Tzu Chi Hospital, Buddhist Tzu Chi Medical Foundation and Tzu Chi University, Hualien 970, Taiwan

**Keywords:** urethra, onabotulinumtoxinA, voiding, therapeutic outcome

## Abstract

Botulinum toxin A (BoNT-A) urethral sphincter injections have been applied in treating voiding dysfunction but the treatment outcome is not consistent. This study analyzed treatment outcomes between patients with different bladder and urethral sphincter dysfunctions. Patients with refractory voiding dysfunction due to neurogenic or non-neurogenic etiology were treated with urethral sphincter 100 U BoNT-A injections. The treatment outcomes were assessed by a global response assessment one month after treatment. The bladder neck opening and urodynamic parameters in preoperative videourodynamic study were compared between successful and failed treatment groups. A total of 80 non-neurogenic and 75 neurogenic patients were included. A successful outcome was noted in 92 (59.4%) patients and a failed outcome in 63 (40.6%). The treatment outcome was not affected by the gender, voiding dysfunction subtype, bladder dysfunction, or sphincter dysfunction subtypes. Except an open bladder neck and higher maximum flow rate, no significant difference was noted in the other variables between groups. Non-neurogenic patients with successful outcomes had a significantly higher detrusor pressure, and patients with neurogenic voiding dysfunction with successful results had higher maximum flow rates and smaller post-void residuals than those who failed the treatment. However, increased urinary incontinence was reported in 12 (13%) patients. BoNT-A urethral sphincter injection is effective in about 60% of either neurogenic or non-neurogenic patients with voiding dysfunction. An open bladder neck during voiding and a higher maximum flow rate indicate a successful treatment outcome.

## 1. Introduction

Voiding dysfunction may result from detrusor underactivity (DU), bladder outlet obstruction (BOO), urethral sphincter hyperactivity, or poor relaxation of the external urethral sphincter (PRES) during micturition. A previous urodynamic study reported DU in 12.4% of men [1] and in 23.1% of women [2] with voiding lower urinary tract symptoms (LUTS). Urethral sphincter hyperactivity was noted in 17.0% of women [3], and PRES in 39.5% of men [1] and 17.6% of women [3] with voiding dysfunction. Voiding dysfunction may be neurogenic or non-neurogenic in origin, with symptoms of dysuria and large post-void residual (PVR) volume, and might result in upper urinary tract deterioration. Recently, botulinum toxin A (BoNT-A) has been applied as an injection into the urethral sphincter to treat voiding dysfunction refractory to medical treatment.

In the beginning, BoNT-A was used to treat patients with severe dysuria due to spinal cord injury (SCI) and detrusor sphincter dyssynergia (DSD) [4]. Reduction of the urethral sphincter hypertonicity through chemical denervation was noted after treatment. Patients usually can urinate more efficiently. BoNT-A has been safely used for treatment of neurogenic urethral sphincter spasticity in patients with DSD due to SCI, Parkinson’s disease, cerebrovascular accidents (CVA), and multiple sclerosis (MS). The treatment outcome was initially reported satisfactorily, with adverse events of the increase of urinary incontinence and incomplete bladder emptying [5].

Because BoNT-A can improve voiding efficiency and detrusor contractility, this treatment has been further applied to treat patients with non-neurogenic voiding dysfunction (NNVD) due to urethral sphincter hyperactivity, PRES, and a non-relaxing urethral sphincter in patients with dysfunctional voiding (DV) or DU [6,7]. After BoNT-A injection, two thirds of patients voided smoothly and had significant decreases in PVR; the voiding derusor pressure was also decreased [7]. Even a dose of 50 U onabotulinumtoxinA could result in excellent and improved results overall in 39% of patients [8]. However, because the therapeutic outcome is not consistent and the therapeutic duration was short, urethral BoNT-A injection for voiding dysfunction remains an off-label treatment. In contrast, BoNT-A detrusor injection has already been licensed for use in patients with neurogenic detrusor overactivity (NDO) or non-neurogenic overactive bladder (OAB) to treat urinary incontinence [9]. Nevertheless, for patients with voiding dysfunction not due to BOO, urethral BoNT-A injections remain an attractive treatment modality for various types of adults with pediatric voiding dysfunctions [10,11].

Although BoNT-A has been used in treatment of voiding dysfunction for a long time, the success rate varies widely and patients might not be satisfied with the treatment outcome. Urethral BoNT-A injection had been shown to decrease voiding pressure in NNVD, but the success rate was not superior to normal saline injection [12]. For patients with Fowler’s syndrome and urinary retention due to urethral sphincter hyperactivity, onabotulinumtoxinA injection could improve subjective and objective parameters [13]. In patients with DU and voiding dysfunction, a 60% success rate can be achieved after urethral BoNT-A injection [14]. Because voiding is a combination of detrusor contraction and relaxation of the bladder outlet structures, including the bladder neck, prostatic urethra (in men), urethral sphincter, pelvic floor muscles, and the urethra, voiding dysfunction might be caused by a different combination of bladder and bladder outlet dysfunctions during voiding. In patients with DU, a powerful abdominal pressure by straining is necessary, while in patients with DV or DSD, adequate relaxation of the urethral resistance is needed to achieve efficient voiding. BoNT-A urethral sphincter injection can reduce urethral sphincter resistance, but this therapeutic effect might not be adequate to restore voiding efficiency in all patients. This study retrospectively analyzed the results of our previously treated patients with voiding dysfunction and compared the therapeutic efficacy between patients with voiding dysfunction due to neurogenic versus non-neurogenic origin, and between different detrusor functions (DU versus non-DU) or urethral dysfunction (DV versus PRES).

## 2. Results

A total of 155 patients underwent their first-time urethral BoNT-A injection for their voiding dysfunction refractory to medical therapies. The patients included 80 with NNVD (22 men and 58 women, aged 66.6 ± 16.9 years) and 75 with neurogenic voiding dysfunction (NVD, 34 men and 41 women, aged 55.5 ± 19.7 years). Successful outcomes were reported in 92 (59.4%) patients, and a failed outcome was noted in 63 (40.6%). Table 1 lists the baseline patient demographics according to their voiding dysfunction subtypes, bladder dysfunction, and urethral sphincter dysfunctions between successful and failed subgroups. We found that the treatment outcome was not significantly different among different voiding dysfunction subtypes.

When we compared the videourodynamic study (VUDS) characteristics between patients with successful and failed outcomes, only the baseline maximum flow rate (Qmax) and an open bladder neck during voiding showed a significant difference between groups (Table 2). In the 92 patients with a successful outcome, 89 (96.7%) had an open bladder neck, whereas of the 63 with failed outcomes, 54 (85.7%) had a tight bladder neck (*p* < 0.001). Patients with a successful outcome had a significantly higher Qmax than those with failed outcome (*p* = 0.031). However, this fact was only observed in patients without DU (7.82 ± 4.97 versus 2.0 ± 2.65 mL/s, *p* = 0.004), but not in patients with DU (5.05 ± 5.15 versus 6.0 ± 5.66 mL/s, *p* = 0.804).

The treatment outcome was also not related to the bladder dysfunction between NVD and NNVD patients (Table 3). Further analysis of the VUDS parameters revealed that in patients with NNVD and non-DU, patients with successful outcomes had significantly higher voiding detrusor pressure (Pdet) than those with failed outcomes (*p* = 0.013), but that was not found in patients with NNVD and DU (*p* = 0.456). In patients with NVD and non-DU, those with successful outcomes had significantly lower first sensation of filling (FSF, *p* = 0.050), smaller cystometric bladder capacity (CBC, *p* = 0.010), higher Qmax (*p* = 0.044), and smaller PVR (*p* = 0.011) than patients with failed outcomes. In patients with NVD and DV, those with successful outcomes (*n* = 28) had a significantly lower FSF and full sensation (FS) and smaller CBC (*p* = 0.008) than those with failed outcomes (*n* = 24). Patients of NVD and DSD and a successful outcome had a higher Qmax (*p* = 0.042) and smaller PVR (*p* = 0.021) than those who failed the treatment. Interestingly, significantly more patients with a successful outcome had an open bladder neck (BN) during VUDS than those with a failed outcome, in any subgroup of the bladder or urethral sphincter dysfunction. (Table 4)

After urethral BoNT-A injection for treatment of voiding dysfunction, increased urinary incontinence was reported in 12 (13%) patients with successful outcomes, mostly occurring during sleep. De novo urinary tract infection was only observed in four (2.6%) patients overall.

## 3. Discussion

The results of this study revealed that BoNT-A urethral sphincter injection can improve voiding efficiency in about 60% of patients regardless of neurogenic or non-neurogenic etiology. Preoperative VUDS provides an important prognostic indicator for successful treatment. Patients with NNVD who had higher voiding detrusor pressure and smaller PVR might benefit more from urethral sphincter BoNT-A injection than those with lower voiding pressure. Patients who were found to have a tight bladder neck during VUDS and the DU patients with a very low Qmax might have less favorable therapeutic outcomes. In addition, patients with non-DU NVD and reduced bladder sensation might not benefit from urethral sphincter BoNT-A injection.

The application of BoNT-A in urology started from urethral sphincter injections for the treatment of DSD in patients with SCI and MS [4]. After that, the treatment was extended to treat DO and urinary incontinence in NVD and NNVD patients [8,15]. Double-blind placebo-controlled studies of therapeutic efficacy of BoNT-A urethral sphincter injection have also confirmed the validity and durability of this treatment in patients with SCI and DSD [4,16]. A 50% reduction of the occurrence of urinary tract infection after urethral sphincter BoNT-A injections for DSD has also been reported in a meta-analysis [17]. Previous studies have shown that urethral sphincter injections with 100 to 200 U of BoNT-A were effective in patients with voiding dysfunction due to MS, CVA, or SCI [8,18]. Patients with chronic CVA and chronic urinary retention might be able to get rid of clean intermittent catheterization (CIC) after the urethral injection of 100 U of BoNT-A [8,19].

Several small cohort studies also confirmed the successful therapeutic results in patients with NNVD and voiding dysfunction at doses of 100 U or 50 U of BoNT-A [7,20,21]. Some patients with NNVD and DU could also have recovery of detrusor contractility after urethral sphincter BoNT-A injections and long lasting therapeutic effects [22]. However, until now, urethral BoNT-A injection for voiding dysfunction remained an off-label treatment. Although urethral BoNT-A injection can result in relaxation of the striated urethral sphincter [12], patients with DU and a tight bladder outlet might not have a successful treatment outcome because the bladder neck cannot open on micturition [14]. The treatment results between patients with NVD and NNVD, or between patients with different bladder contractility and urethral sphincter tonicity have not been compared. In addition, although urethral sphincter BoNT-A injection is effective in improving voiding efficiency (VE) and decreasing PVR, incomplete emptying remains a problem to be solved, and postoperative urinary incontinence is still another de novo issue for women with SCI or MS [8,18,23,24]. Under these considerations, patients might not be completely satisfied with the treatment outcome of urethral sphincter BoNT-A injection [23,25]. Therefore, patient selection is important for a successful treatment.

Urination is a complex interaction with appropriate coordination among the central and peripheral neural controls, sustained detrusor contraction, adequate bladder neck relaxation, and complete relaxation of the external sphincter and pelvic floor muscles. With one or more defects of those micturition mechanisms, patients may develop NVD or NNVD. Patients with DSD or DV usually cannot achieve efficient voiding due to hypertonicity of the external sphincter. Patients with DU who use abdominal pressure to void might have voiding difficulty and incomplete bladder emptying due to a tight bladder neck or a non-relaxing urethral sphincter or pelvic floor. Patients with detrusor hyperreflexia (DHIC) might have significant PVR because of low detrusor contractility without any anatomical BOO. In the era of BoNT-A, although theoretically the urethral sphincter BoNT-A injection might produce benefits by reducing bladder outlet resistance in voiding dysfunction, the unrealistic expectations of BoNT-A urethral sphincter injections often results in failed treatment in patients not suitable for this treatment [23].

In this study, we found that an open bladder neck during VUDS is essential for successful BoNT-A injection in patients with NVD or NNVD. A tight bladder neck during voiding is an unfavorable prognostic factor for the successful outcome of BoNT-A treatment. If the bladder neck cannot be opened, urine output will be inhibited at this gate. On the contrary, after transurethral incision of the bladder neck, patients with DSD or DU usually can urinate by abdominal straining or detrusor contraction after the urethral BoNT-A injection. However, urinary incontinence might be a bothersome problem. Before urethral sphincter BoNT-A injection, patients should be informed about this potential adverse event. This adverse event is also the reason why many NVD patients finally select detrusor BoNT-A injections and periodic CIC for the solution of their voiding problem [23].

BoNT-A urethral sphincter injection results in decreased urethral pressure, increased Qmax, decreased PVR, and a reduction of autonomic dysreflexia in NVD patients with DSD due to SCI or MS [4,5,12,25]. The external sphincter hypertonicity might have different severity; therefore, injection of 100 U of BoNT-A might not be adequate for an efficient voiding in high grade DSD. In addition, because patients with NVD and DSD also have uninhibited DO, an increased urinary incontinence grade might develop after effective urethral sphincter BoNT-A injections [26].

NNVD due to DV is difficult to treat because the actual pathophysiology has not been elucidated and the only known LUTD is dysregulated urethral function with spastic or a non-relaxing external urethral sphincter during voiding [27]. DV results in voiding symptoms: Slow stream and large PVR. Therefore, attempts to reduce the hypertonicity or hyperactivity of the urethral sphincter by medication, and resume spontaneous voiding, always results in failure. It is also postulated that the psychologic voiding dysfunction due to anxiety or depression might be a cause of a low detrusor contractility and non-relaxing urethral sphincter through inhibiting detrusor contraction [28]. BoNT-A urethral sphincter injection can reduce urethral resistance but does not have an effect on the psychological insult; therefore, the successful outcome was only observed in 60% of patients with DV. If the bladder neck is not open during voiding, patients with DV might have more difficulty urinating.

DU may result from neurogenic, myogenic, obstructive, or idiopathic etiology. DU patients need to use abdominal pressure to void or depend on CIC. A sustained abdominal pressure is necessary to efficiently empty the bladder [29]. If the bladder outlet resistance is high at the level of bladder neck or urethral sphincter, patients need higher abdominal pressure to overcome the resistance, and therefore, they might not able to void efficiently. If the bladder neck is not open during voiding, urethral BoNT-A injections to the urethral sphincter might not be successful. Therefore, if VUDS shows a tight bladder neck, patients with DU and voiding dysfunction should undergo transurethral incision of the bladder neck first, otherwise, BoNT-A urethral sphincter injections might fail. In addition to an open BN, an adequate abdominal pressure is necessary for patients with DU who wish to void spontaneously after urethral BoNT-A injection. 

Normal bladder sensation is another important factor for an efficient urination. The sensory afferents from the bladder urothelium and detrusor are essential parts of the voiding reflex circuit. Patients with DU might have reduced bladder sensations of filling and fullness [30]. The deficit of the bladder sensation will cause difficult initiation of voiding, as well as inefficient bladder emptying when the bladder has not been completely emptied [31]. Therefore, although the urethral resistance has been reduced after the BoNT-A injection, patients with DU and reduced bladder sensation might still have a poor outcome after treatment, especially in patients with NVD [32]. For these patients timed voiding and instruction of correct usage of abdominal pressure to void are mandatory after urethral sphincter BoNT-A injection in patients who have DU and reduced bladder sensation.

A limitation of this study is the mixed patient cohort. We aimed to find predictive factors for patients with different bladder and urethral sphincter dysfunctions. Interestingly, the success rates were similar between NVD and NNVD, and between different bladder or urethral sphincter dysfunctions. Treatment of voiding dysfunction is not an easy task. The possible causes of treatment failure have several different aspects [27]. Identification of the underlying causes of failure may improve the success rate after urethral sphincter BoNT-A injection. In this regard, VUDS before urethral BoNT-A treatment is mandatory to assess the vesicourethral dysfunction in patients with NVD and NNVD. Careful evaluation of the bladder neck opening and a higher Qmax at baseline may provide predictive value for a successful BoNT-A treatment outcome. However, urinary incontinence might be a de novo adverse event after urethral sphincter BoNT-A injection. Patients who are planning to undergo urethral sphincter BoNT-A injection for voiding dysfunction should be fully informed of the limited therapeutic efficacy and the possible adverse event of urinary incontinence before treatment.

## 4. Materials and Methods

This study retrospectively analyzed consecutive patients with voiding dysfunction who were refractory to medical treatment and received 100 U of BoNT-A (onabotulinumtoxinA, Allergan, Irvine, CA, USA) via urethral sphincter injection from 2011 to 2019. All patients underwent VUDS and cystoscopy to identify the underlying pathophysiology of lower urinary tract dysfunction before the BoNT-A injections. Patients with anatomical BOO, such as urethral stricture, bladder neck obstruction, or benign prostatic obstruction were excluded from the study. Only patients with DU who required spontaneous voiding by abdominal straining, had voiding dysfunction due to non-relaxing urethral sphincter, had spinal cord lesion(s) and DSD, and had DV were included in the final analysis. This study was approved by the ethics committee of the institution (IRB 105-151-B). Date of approval: 29 December 2016 to 14 December 2017. Informed consent was waived by the committee due to the retrospective nature of the study. All patients were informed of the potential adverse events after BoNT-A injection before treatment.

The treatment was performed in the operating room under light intravenous general anesthesia. A total of 100 U BoNT-A was given via transurethral sphincter injections [8]. One vial of 100 U onabotulinumtoxinA was reconstituted with normal saline to 4 mL, making the concentration equivalent to 25 U per mL. One mL of BoNT-A solution was injected into the urethral sphincter at the 3, 6, 9, and 12-o’clock positions transurethrally in men, and transcutaneous injection to the urethral sphincter along the urethral lumen at 1, 4, 7, and 10 o’clock positions of the sides of the urethral meatus in women.

A Foley catheter was indwelled overnight after BoNT-A injections and the voiding condition was requested to be reported at the out-patient clinic. The effect of BoNT-A on the urethral sphincter function appeared about 2–3 days after the treatment. The maximum therapeutic effect would reach about 2 weeks after BoNT-A injection [8]. Patients with DU and large PVR were instructed to void by Crede maneuver or abdominal straining, and CIC was recommended instead of an indwelling Foley catheter. The Qmax and PVR were measured and CIC was continued until PVR was reduced to less than 50% of the voided volume. Antibiotics were routinely given for 3 days to prevent urinary tract Infections and all medications for reduction of urethral resistance were discontinued after BoNT-A injections.

VUDS was performed in all patients at baseline. The VUDS parameters included the opening or closed bladder neck during voiding cystourethrography, the FSF, CBC, Qmax, Pdet, PVR, intra-abdominal pressure to void (in patients with DU), and VE were recorded. The terminology used in this study was based on the recommendations of the International Continence Society [33]. Patients with voiding dysfunction caused by SCI, CVA, MS, or peripheral neuropathy were categorized as having NVD, otherwise, patients were considered as NNVD. For analysis of the treatment outcome, the bladder dysfunctions were categorized into hypersensitive bladder, DO, DHIC, and DU groups. The urethral sphincter dysfunctions were caegorized as DV, DSD, and PRES, according to the electromyographic characteristics and images during the voiding phase.

The treatment outcome was assessed by self reported satisfaction and the change of the VE at 1 month after the BoNT-A injection. Patients were requested to report their global response assessment (GRA) after BoNT-A injection as: Excellent (+3), markedly improved (+2), mildly improved (+1), no change (0), or worsened (−1). Treatment success was defined as a report with GRA ≥ 1 and the VE ≥ 50% after BoNT-A injection. If patients had VE of less than 50% and GRA equaled to 0 or −1, they were considered to have had failed treatment. Adverse events after BoNT-A injections were also recorded and appropriate treatments were given if needed.

Continuous variables were expressed as means ± standard deviations (SD). Categorical data were presented as numbers and percentages (%). We used chi-square test for categorical variables, and the Wilcoxon rank sum test for continuous variables to determine the *p*-values between successful and failed subgroups for statistical comparisons. All statistical assessments were two-sided and considered significant at a *p* < 0.05. All calculations were performed using SPSS for Windows, version 16.0 (SPSS, Chicago, IL, USA).

## 5. Conclusions

BoNT-A urethral sphincter injection is effective in approximately 60% of patients with voiding dysfunction due to NVD or NNVD, refractory to conventional medical treatment. Careful VUDS interpretation of bladder neck opening and measurements of a higher detrusor or abdominal pressure enabled us to select candidates suitable for urethral sphincter BoNT-A treatment.

## Figures and Tables

**Table 1:** Treatment outcome according to patient characteristics at baseline.

VD Characteristics		N	Successful Outcome	Failed Outcome	*p* Value
VD Subtype	Idiopathic VD Neurogenic VD	80 75	50 (62.5%) 42 (56%)	30 (37.5%) 33 (44%)	0.255
Gender	Male Female	54 99	32 (59.3%) 60 (60.6%)	22 (40.7%) 39 (39.4%)	0.445
Bladder Function	DO DU DHIC HSB	57 59 25 14	36 (63.2%) 34 (57.6%) 15 (60%) 7 (50%)	21 (36.8%) 25 (42.4%) 10 (40%) 7 (50%)	0.819
Sphincter Function	DV DSD PRES	115 237	70 (60.9%) 14 (60.9%) 8 (47.1%)	45 (39.1%) 9 (39.1%) 9 (52.9%)	0.550

VD, voiding dysfunction; DO, detrusor overactivity; DU, detrusor underactivity; DHIC, detrusor hyperreflexia and inadequate contractility; HSB, hypersensitive bladder; DV, dysfunctional voiding; DSD, detrusor sphincter dyssynergia; PRES, poor relaxation of external sphincter.

**Table 2:** The video-urodynamic (VUDS) characteristics between patients with successful and failed treatment outcomes.

VUDS Findings		Successful Outcome	Failed Outcome	*p* Value
		(*n* = 92)	(*n* = 63)	
Age (years)		62 ± 18.8	64.7 ± 20.3	0.692
First sensation of filling (mL)		140.1 ± 73.0	132 ± 74.6	0.792
Cystometric bladder capacity		338 ± 142	267 ± 135	0.153
Detrusor pressure (cmH_2_O)		25.2 ± 25.2	39.0 ± 40.7	0.344
Abdominal pressure (cmH_2_O)		36.7 ± 36.3	34.3 ± 38.1	0.860
Maximum flow rate (mL/s)		6.76 ± 5.19	2.89 ± 3.52	0.031
Post-void residual volume (mL)		218 ± 5.19	202 ± 151	0.799
Open bladder neck		89 (96.7%)	9 (14.3%)	< 0.001
DU	Open BN	34 (94.4%)	2 (5.6%)	< 0.001
	Tight BN	2 (7.7%)	24 (92.3%)	
Non-DU	Open BN	55 (88.7%)	7 (11.3%)	< 0.001
	Tight BN	1 (3.2%)	30 (96.8%)	

DO, detrusor overactivity; DU, detrusor underactivity; BN, bladder neck.

**Table 3:** The video-urodynamic (VUDS) characteristics of voiding dysfunctions between patients with successful and failed treatment outcomes.

VUDS Findings		Successful Outcome (*n* = 92)	Failed Outcome (*n* = 63)	*p* Value
**Bladder Function**				
Non-neurogenic	DU	19 (67.9%)	9 (32.1%)	0.316
	Non-DU	31 (59.6%)	21 (40.4%)	
Neurogenic	DU	16 (48.5%)	17 (51.5%)	0.177
	Non-DU	26 (61.9%)	16 (38.1%)	
**Sphincter Function**				
Non-neurogenic	DV	46 (62.2%)	28 (37.8%)	0.598
	PRES	4 (66.7%)	2 (33.3%)	
Neurogenic	DV	28 (53.8%)	24 (46.2%)	0.379
	DSD	14 (60.9%)	9 (39.1%)	

DU, detrusor underactivity; DV, dysfunctional voiding; DSD, detrusor sphincter dyssynergia; PRES, poor relaxation of external sphincter.

**Table 4:** Comparison of the baseline video-urodynamic parameters between patients with successful and failed treatment outcomes in different bladder and urethral sphincter dysfunctions.

Voiding Dysfunction Subtypes		FSF/FS (mL)	CBC (mL)	Pdet (cmH_2_O)	Pabd (cmH_2_O)	Qmax (mL/s)	PVR (mL)	BN Condition	
								Tight	Open
**NVD-non-DU (*n* = 42)**	S F *p*	111 ± 52.3 157 ± 80.7	262 ± 129 381 ± 149	32.1 ± 26.7 26.0 ± 22.2	18.9 ± 18.8 26.0 ± 26.1	7.22 ± 6.03 3.60 ± 3.94	162 ± 124 285 ± 174	1 (8.3%) 11 (91.7%)	26 (86.7%) 4 (13.3%)
		0.050	0.010	0.456	0.316	0.044	0.011	0.000	
**NVD-DU (*n* = 33)**	S F *p*	182 ± 104 230 ± 91.6	384 ± 150 431 ± 124	6.06 ± 8.65 2.53 ± 4.03	71.3 ± 47.0 68.2 ± 35.9	4.50 ± 4.26 3.76 ± 3.62	253 ± 143 329 ± 161	0 (0.0%) 16 (100%)	16 (94.1%) 1 (5.9%)
		0.174	0.336	0.139	0.837	0.596	0.164	0.000	
**NNVD-non-DU (*n* = 53)**	S F *p*	132 ± 52.8 141 ± 57.0	350 ± 140 343 ± 109	39.6 ± 28.9 23.2 ± 17.5	29.4 ± 32.6 39.1 ± 29.7	8.00 ± 4.08 6.23 ± 4.57	176 ± 137 228 ± 129	1 (5.3%) 18 (94.7%)	30 (88.2%) 4 (11.8%)
		0.576	0.836	0.013	0.272	0.144	0.168	0.000	
**NNVD-DU (*n* = 27)**	S F *p*	161 ± 72.4 180 ± 79.0	373 ± 115 360 ± 171	7.85 ± 8.20 7.86 ± 7.56	40.0 ± 27.3 34.3 ± 40.7	4.90 ± 5.57 3.57 ± 3.87	275 ± 171 274 ± 182	3 (33.3%) 6 (66.7%)	17 (94.4%) 1 (5.6%)
		0.563	0.815	0.998	0.679	0.567	0.993	0.002	
**NVD-DV (*n* = 52)**	S F *p*	145 ± 66.4 215 ± 96.7	323 ± 151 450 ± 149	19.9 ± 25.3 9.89 ± 14.9	38.5 ± 35.4 49.4 ± 37.5	6.04 ± 6.33 3.50 ± 3.43	202 ± 130 328 ± 177	1 (5.9%) 16 (94.1)	25 (92.6%) 2 (7.4%)
		0.013	0.008	0.139	0.329	0.129	0.016	0.000	
**NVD-DSD (*n* = 23)**	S F *p*	113 ± 102 168 ± 83.8	261 ± 134 344 ± 102	29.7 ± 24.8 21.8 ± 25.6	31.4 ± 42.5 44.6 ± 43.9	7.33 ± 3.74 3.91 ± 4.39	157 ± 124 289 ± 148	0 (0.0%) 8 (100%)	15 (83.3%) 3 (16.7%)
		0.152	0.099	0.435	0.450	0.042	0.021	0.000	
**NNVD-DV (*n* = 74)**	S F *p*	144 ± 63.4 149 ± 66.0	361 ± 132 349 ± 127	28.1 ± 28.0 20.9 ± 17.0	31.4 ± 29.1 40.6 ± 32.7	6.92 ± 4.96 5.77 ± 4.48	213 ± 160 237 ± 135	4 (14.8%) 23 (85.2%)	45 (93.8) 3 (6.3%)
		0.735	0.709	0.175	0.218	0.327	0.514	0.000	
**NNVD-NR (*n* = 6)**	S F *p*	138 ± 17.7 183 ± 38.9	304 ± 65.1 305 ± 134	5.00 ± 0.00 0.00 ± 0.00	85.0 ± 35.4 12.5 ± 10.6	3.50 ± 2.12 1.00 ± 1.41	250 ± 70.7 350 ± 212	0 (0.0%) 1 (100%)	2 (66.7%) 1 (33.3%)
		0.275	0.993	0.218	0.109	0.300	0.592	1.000	

FSF, first sensation of filling; FS, full sensation; CBC, cystometric bladder capacity; Pdet, detrusor pressure; Pabd, abdominal pressure; Qmax, maximum flow rate; PVR, postvoid residual; BN, bladder neck; DO, detrusor overactivity; NVD, neurogenic voiding dysfunction; NNVD, non-neurogenic voiding dysfunction; DU, detrusor underactivity; DV, dysfunctional voiding; DSD, detrusor sphincter dyssynergia; NR, non-relaxing external sphincter; S, successful outcome; F, failed outcome.
